# Book Reviews

**Published:** 1808-01

**Authors:** 


### CRITICAL ANALYSIS
### OF THE
### RECENT PUBLICATIONS
### ON THE
### DIFFERENT BRANCHES OF PHYSIC, SURGERY, AND MEDICAL PHILOSOPHY.

---

*The Lectures of Boyer upon the Diseases of the Bones: arranged into a Systematic Treatise.* By A. RICHERAND, Professor of Anatomy and Philosophy, and Principal Surgeon to the Northern Hospital at Paris. Translated from the French by M. FARRELL, M. D. In 2 vols. 8vo.

HOWEVER unwilling we may be to acknowledge it, every reference to French authors proves that they have been in practical surgery the masters of all Europe. It is true, their erroneous physiology has retarded the advantage which their sagacious precepts would otherwise have produced; but our improvement in physiology has usually confirmed the propriety of their lessons. The present work is infinitely more comprehensive than any other of the kind, containing, at the same time, all the modern improvements, and some that, if they are well known in England, we confess, have escaped our notice. The mere English reader will be particularly surprized at finding those nice distinctions of Mr. Hunter, between ulceration, caries, and exfoliation, accurately ascertained, though under different names. The process of nature in each is marked. It is true, the great apprehension of hectic from absorption of matter, may in some instances have warped the author's reasoning; but in these there is no danger, lest the English reader should be misled, whilst he will meet with ample instruction in some of the most troublesome cases that occur in practice.

In reviewing this work, we shall follow our usual custom of showing, in as few words as possible, the contents of the volumes, offering specimens of the style in which they are executed, and our own remarks as we proceed.

A short Introduction is prefixed, in which it is remarked, that the diseases of the bones are similar to those of the soft parts, allowing only for the slowness of action, which must follow from the smallness of their vessels, and the deposition of phosphate of lime. The old arrangement is adhered to, by which the diseases of these parts are divided into two orders; the first, comprehending whatever affects each bone singly; the second, their diseases in the joints, and in their points of contact one with another; admitting at the same time, that under the latter division the substance of the bones is often affected from the same cause, or from a continuity of the same affection.

Under the first order are contained fractures, wounds, exostosis, necrosis, necrosis and caries, the ricketty softening of the bones, their friability, and that morbid state known by the name of spina ventosa or osteosarcoma. The second order embraces sprains, luxations, dropsy of the articulations, the diseases arising from preternatural substances generated in the articulations, white and lymphatic swellings, and anchylosis. On each of these we shall offer a few remarks.

In considering the first order we cannot help wishing, as these diseases are so similar to others in the soft parts, that they had been called as much as possible by the same names. The introduction of the word necrosis is an unnecessary affectation, never sanctioned by Cullen, though since adopted by some of the writers of that professor's school. Necrosis is, properly speaking, the mortification of a part, or the whole of a bone. Caries is the *ulcer mali moris*, arising from what we commonly denominate scrofula, when it cannot be traced to any of the morbid poisons: either of these, as well as the effects of wounds, may end in exfoliation. Exostosis is a general term for a partial enlargement of the bone, so that osteosarcoma might have been included in this division:—Of this we shall say more when those chapters come before us.

Fractures being the most common injuries of bones, and requiring a promptness and decision in the mode of treatment, as well as the most practical knowledge, are first considered. Their symptoms and treatment are very accurately detailed, and though in every respect they do not exactly agree with our English practice, yet for that reason we could wish they were universally attended to by the surgeons of this country. It is well known, that stubborn cases sometimes occur, in which it is desirable to vary our practice, and the knowledge of all the little improvements suggested by the French, however unnecessary on common occasions, may be useful on these. There may even be circumstances under which the great improvement of Mr. Pott, in placing the lower limb half bent, and on its side, may be objectionable; but we confess our surprise at finding a contrary practice so general on the continent, if it really is so. We well recollect, with how much satisfaction Professor Scarpa, when in the country, used to claim the merit of having introduced Mr. Pott's method into Italy. We shall transcribe what is here said on the subject, as well as an improvemement on the eighteen-tailed bandage, though, probably, that improvement has been common in the London hospitals since we *walked* those rounds of practical knowledge.

"Surgeons were for some time divided in their opinions on the best position. Pott has advised the limb to be kept half bent; which position, he says, has the advantage of giving to the muscles which surround an articulation, an equal and moderate degree of tension; whereas, if the member be placed straight, some of these muscles will be much extended, whilst others are as much relaxed. The latter position, however, is that which is generally preferred.

"Demi-flexion is the most natural position; it is that which our our limbs spontaneously assume during sleep, and has for that reason been recommended both by Galen and Hippocrates: but a limb half bent, is not solidly fixed, and changes frequently its situation, by numerous involuntary motions, which may be occasioned by dreams or pain. This position has, besides, this great inconvenience, that during the treatment the length of the fractured limb cannot be compared with that of the opposite side, nor can it therefore be known if the fracture be well set, and the apparatus well applied; and in truth, demi-flexion becomes at length as painful as the extension at full length. The advantages of the former have therefore been a little exaggerated by Pott, as well as the disadvantages of the latter, which alone is now used in France, and generally adopted in foreign countries.

"In whatever position the limb is placed, most perfect repose is absolutely necessary, particularly in the commencement; for if the fractured pieces be moved one upon the other, nature cannot effect their re-union, which would therefore be retarded or totally prevented, if the friction of the surfaces were frequent, and suffered to continue long; in which case an articulation would be formed in the situation of the fracture, and consequently the patient remain disabled for ever after.

"It is necessary to apply proper bandages and other apparatus, without which the position given to the member, however good, would be insufficient; because, without these, the involuntary motions which are inevitable, those which are produced by pain, and those again which are rendered necessary by our natural wants, would certainly, without that precaution, disturb more or less the just relative position of the fractured pieces, which even the depressions and inequality of the bed would affect, if not guarded against.

"Bandages had been for a long time considered as the most effectual means of retaining the fragments in just contact; but it is easy to prove, that bandages, however contrived, can have but little, or absolutely no effect for this purpose. We shall examine successively, those which have been in use, viz. the roller, eighteen-tailed bandage, and that of Scultet, composed of separate pieces.

"The first ought to be long enough to cover the whole limb, three inches broad, and rolled up in one. It is applied, by drawing first three folds of it over the fractured parts; it is then made to descend to the extremity of the limb, in such a manner, as that each roll shall cover a part of the preceding; it is made to ascend again in like manner to the situation of the fracture, when three folds more of it are applied; after which the superior part of the limb is covered, in the same manner as the inferior; and if the bandage be long enough, it may be again rolled on downwards. Let us suppose this bandage applied to a fracture of the middle part of the femur, or humerus: it is plain that those parts of it which are applied on one of the fragments alone, are absolutely of no effect, and that that part of it only which comprehends both fragments, can contribute to keep them in contact. But in order to understand how extremely trifling its effect must be, it is sufficient to remark, that, as it is but three inches broad, it can include only an inch and a half of each fractured portion; and that this very trifling power is still farther diminished, by the greater or less quantity of soft parts which intercept its action.

"In this respect, the eighteen-tailed bandage is preferable to the former. It is composed of three pieces of linen, equal in length to the member, and broad enough to pass once and a half round the limb. These pieces are sewed together by a seam, which runs from one extremity to the other; and afterwards cut each into three; so that the whole is composed of eighteen pieces, nine at each end. The bandage thus composed, being moistened, is extended under the limb, and the middle piece on each side is first applied on the situation of the fracture, then the superior ones, and afterwards the inferior, and so successively with the middle and inferior pieces. The six middle pieces of this bandage act with more efficacy on the fracture, than the folds of the former bandage; because, being much broader, they encompass a greater part of it. It has this other advantage over the former bandage, that its application is easier, and does not require that the limb should be kept raised, nor exposed to many motions, which are always hurtful.

"Scultet's bandage is composed of as many pieces of three inches broad each, as are necessary to cover the whole length of the member, in lapping two thirds over one another. It is composed of a piece of linen, of three times the length of the limb, and broad enough to pass once and a half round the limb; it is to be cut according to its breadth, in pieces of three inches broad each: this done, the pieces are extended under the member, one covering three parts of the other in proceeding from the inferior part. This bandage, like the two former, acts only by the pieces which encompass at once the contiguous parts of the two fragments; it is preferable, however, in the following respects.

"It contributes as much as the others to maintain the fragments in their just position; it compresses sufficiently the member, and prevents any œdema; it is, in this respect, preferable to the eighteen-tailed bandage, the parts of which not passing one on the other, do not compress all the parts equally; whence it happens, that those parts which correspond to the edges of the pieces become œdematous.

"This bandage can be taken off, and reapplied, without moving the limb, in which it is far preferable to the first-mentioned bandage, the disadvantage of which in that respect has been already pointed out. The eighteen-tailed bandage cannot be so conveniently renewed as that of Scultet, because, when any part of it is soiled by purulent matter, or any other cause, it is necessary to remove it entirely, and apply another; whereas any particular piece of that of Scultet may be changed, and a new one applied in its place, which may be done without moving the member, by fastening ing the new one to the extremity of the old, and drawing it into its proper situation at the same time that this latter is taken away. It ought, therefore, to be preferred to the two former, except in cases of simple fracture of the superior extremities.

"Although bandages may be of no great use for keeping the broken pieces in their proper position, yet they are useful for supporting topical applications, and preventing œdema of the limb; they are still further useful in benumbing the muscles, and in preventing their contraction."

We would not be supposed to offer the above as a specimen of the best practical remarks contained in the work. On the contrary, if there is any thing more objectionable than the rest, we think it may be met with in our extract. We confess ourselves so much attached to Mr. Pott, that we neither approve of bandaging so tight as to benumb muscles, nor to prevent œdema. From such attempts we fear still worse consequences might happen. But it is right to see the whole of a question; and the general success allowed to French surgeons may teach us, that if such is their practice, it is less inconvenient than we have been taught to believe. In the treatment of the fracture of the olecranon and patella, there is some novelty, and some questions are candidly discussed, on which practitioners are not yet unanimous. All the other fractures are accurately described, and the French mode of treating them is on some occasions varied in such a manner as to furnish useful hints to the English reader.

A chapter follows, on "wounds and denudations of the bones; others, on necrosis, caries, exostosis, and osteosarcoma." These, which conclude the first volume, we have already noticed very slightly. We shall only add, as Mr. Hunter's lectures on these subjects have not been published, we know of no English author so comprehensive on these important and intricate inquiries Not only might the term osteosarcoma have been made generic for all kinds of soft intumescence; but even then the divisions should have been more minute. We are aware of the difficulty of describing them all, and it is but justice to the author to admit that he has often acknowledged the insufficiency of his art. It might have answered some practical advantages if a distinction had been attempted between such as are curable and such for which we have no remedy but amputation. With all these exceptions, it must be admitted, that the practical remarks are numerous and valuable. Even under the division of spina bifida, the English reader will be surprized to find many useful and new observations.

The second volume continues the diseases of the bones, through two more chapters, comprehending rickets and the fragibility of the bones. These are well described, and the practical remarks attached to them are very useful; but the first depending on the general state of the health can only be relieved by an attention to it, and the last being for the most part connected with old age, scarcely admits of any remedy. Of this the author is so well aware, that that he very properly attends more to the accurate history of the diseases than to the remedies which have been suggested from theoretic opinions of their cause.

An useful chapter on sprains, follows, which introduces the subject of luxation. This is first considered in a general view, and afterwards traced through every joint in the human body with much accuracy, and a copious view of all the means of reduction hitherto attempted or proposed.

The diseases of the joints are next considered; the first of these, called hydrarthus, or dropsy of the joints, is, as the author observes, almost confined to the knee, and is distinguished from the white swelling by the attendant pain and inflammation. Its cause is stated to arise from inflammation, so gradual as only to produce more than the usual secretion of cynovia and effusion of lymph. As the last mode of cure, the author is not afraid of proposing plunging a trochar into the joint, with every necessary caution to exclude the air, and prevent excessive inflammation.

The formation of foreign bodies in the articulations is next considered. On this chapter we cannot help making two or three remarks. Whilst the names of Morgagni, Desault, Fourcroy, and others are introduced, we could not help expecting to see something of our British surgeons, who have so well explained the nature of these substances. But of this explanation the author seems perfectly ignorant. We are confirmed in this opinion, because he concludes his Inquiry by acknowledging, that none of the causes to which the disease is ascribed are satisfactory. In the treatment, he advises the operation proposed by our English surgeons, and we are not certain that his plan is not an improvement on them. In order that the opening through the integuments and the capsular ligament should be in different places, and that the whole should be completed by a single incision, he advises that the operator and his assistant should draw the skin in a direction over the tumour, different from its natural position. If this is the English method, it is indeed conceivable that each nation may have introduced it without the knowledge of the other. Still we are surprized that nothing is said of Mr. Hunter's theory of the origin of these substances, from extravasated blood retaining its life, becoming organized and taking up a structure similar to the surrounding parts. This omission is the more remarkable, because we are told that many of these substances "have vessels and evident marks of organization." We suspect that the only cause of this omission must be looked for in the obscurity of Mr. Hunter's language, which not being always intelligible to his countrymen, may still more puzzle a foreigner. We have indulged ourselves with these conjectures, which we shall now conclude the more readily, as the mode of treatment is similar in both nations.

A chapter follows, on wounds in the articulations, which it is now well known are not so disastrous as was apprehended by the ancients. Several cases are given, in which wounds in these parts by by sharp cutting instruments healed by the first intent. The author is ready to admit with the old writers, that the more serious cases arise from the admission of air; but we should rather impute it to the small powers of life in these parts, which render them incapable of supporting high inflammatiou. The practical inference is, however, the same in each; viz. to bring the lips of the wound together as speedily and as accurately as possible, and to admit no extraneous substance.

On the white swelling of the joints the author is very minute, and brings forward all that has been said by others, as well as several practical remarks of his own. But we neither subscribe to his physiological opinions, nor do we find any thing very new in his proposed treatment. Some useful remarks follow, on anchylosis; and a concluding chapter, on "the deviations of bones." The means of preventing and correcting the deformity arising from them is well worth perusing, though some of the instructions are not new to the English reader.

A copious analytical index is annexed to the whole, which the reader will find particularly useful in a book of reference. On the whole, we cannot but recommend this work as an useful appendage to a surgical library, especially for the country practitioner, who has not always the opportunity of being assisted by men in constant practice.

---

*A Treatise on Hernia: being the Essay which gained the Prize, offered by the Royal College of Surgeons in* 1806. *Illustrated with Plates.* By Wm. Lawrence, Member of the College, and Demonstrator of Anatomy at St. Bartholomew's Hospital. 8vo.

In a short preface, the author observes, that neither the intention of the college nor the state of the art, requires a complete treatise on hernia under every appearance—that Pott and Richter have already furnished such a performance; and that the masterly eloquence of the former would make any attempt to follow him not only useless, but degrading by comparison. The college have confined the subject of the proposed essay to the *treatment* of hernia. In addition to this, the author has thought it right to be very particular in his anatomical description of the parts concerned in the disease and in the operation, because these have hitherto been considered by systematic writers as already well known to their readers.

That our author had opportunities of ascertaining all these particulars, cannot be questioned, when we consider his situation in the anatomical school at St. Bartholomew's: that his abilities and industry were equal to them will not be doubted by those who know him; and the general reader will be satisfied by the manner in which the work is executed. It is hardly necessary to add, that the original paper is much enlarged, in order to render it more extensively useful to the learner or young practitioner.

The first chapter contains the description of hernia; its causes; symptoms; of reducible hernia; of strangulated hernia; different species of strangulation, and prognosis of each. The first section of this chapter, pointing out the intention of the work, we shall transcribe in the author's own words.

"The passage of any of the abdominal viscera, from the cavity in which they are naturally contained, into a preternatural bag, formed by the protrusion of the peritoneum, constitutes a *hernia*, or *rupture*, according to the most common acceptation of these terms. The protruded portion of the peritoneum is called the *hernial sac*. For the more particular description of this part, which is a subject of great importance, I refer the reader to the chapter on the anatomy of inguinal hernia.

"A hernia generally causes an external tumour, which is named, either according to its situation in the body, or from the parts which it contains. The groin, scrotum, labia pudendi, bend of the thigh, and navel, are the most frequent seats of these swellings; the omentum and intestines their most common contents.

"When the protruded viscera have descended through the abdominal ring, without passing further than the groin; or when they are contained in the labium of the female, the case is called a *bubonocele*, or *inguinal hernia*: when they have passed into the scrotum, it is termed an *oscheocele*, or *scrotal rupture*; and if they are in contact with the testis, it is distinguished by the epithet *congenital*. The *crural*, or *femoral hernia*, is that which takes place under Poupart's ligament; and the *exomphalos*, or *umbilical* rupture occurs at the navel.

"The names *enterocele* and *epiplocele*, which are equivalent to *intestinal* and *omental* rupture, are employed according as the swelling contains intestine or omentum alone: where both these parts are found in the same tumour, it forms an *entero-epiplocele*.

"These, which are by far the most frequent forms of the complaint, are all that I propose to treat of in the present work.

"So long as the viscera descend and return freely, the complaint is said to be in a *reducible* state. When, after long residence in the tumour, they have either increased so much in bulk, or have contracted such adhesions to each other, or to the hernial sac, as to become incapable of being returned, although they experience no pressure from the ring, it is termed *irreducible*. An incapacity of reduction, arising from stricture in the opening through which the viscera have descended, brings the disease into the *strangulated* or *incarcerated* state."

Under the second section, *the causes of hernia* are well enumerated and described. The author has very properly omitted many of the incidents enumerated by systematic writers; but it would have been better to have omitted the *epithet* with which the causes assigned by Richter are introduced. The symptoms of strangulation and their different species are admirably characterized: the prognosis, unless that it is too short, is unexceptionable. Under the treatment of reducible hernia, is a very useful dissertation tation on the construction and use of trusses. A short chapter follows, on the treatment of ruptures, in which, though no strangulation has taken place, yet reduction is impracticable from adhesions or any other causes.

The fourth chapter is on the treatment of strangulated hernia. The indication of cure, says our author, is to liberate the parts from stricture, and replace them in their natural situation. No one will doubt this: but if the symptoms of incarceration are inflammation, spasm, and constipation, these symptoms will require immediate attention, and probably the chance of success from the attempt at reduction, may in some cases be greater if attention is paid to them in the first instance. As however there cannot be any great difference of opinion in a practical view of such cases, we think the note might have been omitted. As the disease must require different treatment, according to the situation of the patient, his age, and other circumstances, the author very properly allots a distinct section for each means of relief. The first is directed to the taxis, or order in which the attempts at reduction should be made. When this is found impracticable, each auxiliary is commented upon; viz. blood letting, purgatives, tobacco clysters, antispasmodics, cold bath, cold topical applications, and warm applications. Tobacco clysters, as a remedy peculiarly powerful, and requiring most attention, occupy the largest portion of this section. Their effect, it is justly observed, is not confined to a mere purgative quality, other purgative clysters proving for the most part very ineffectual; but the general influence on the system proves highly favourable to the relaxation of the parts which form the stricture. This chapter closes with many useful practical remarks, particularly on the importance of prompt decision in the application of every remedy, and most of all, on the danger of deferring the operation too long.

The fifth chapter, *on the anatomy and symptoms of inguinal hernia*, contains every necessary practical remark. The sixth, on the operation for the strangulated hernia in that part, is divided into three sections. The first describes the opening of the hernial sac; the second, the incision of the stricture; and the third enumerates every possible difficulty that may embarrass the operator with very useful directions under each. A separate chapter contains the treatment of the omentum.

The eighth chapter is on the treatment of hernia, when the intestine is mortified. This is a very useful chapter to the young practitioner in particular, who may be led by the writings of some highly respectable authors to fancy themselves called upon to perform strange operations, and that they are answerable for omitting what has rarely been attempted without danger, and perhaps never with any advantage. Two cases are subjoined, affording each of them very useful practical reflexions.

The ninth chapter, *on the symptoms, anatomical description, and treatment*

*treatment of femoral hernia*, commences with some remarks so judicious, that we shall transcribe a part.

"The femoral hernia is formed by a protrusion of some of the abdominal contents under the inferior margin of the external oblique muscle: and the swelling is situated towards the inner part of the bend of the thigh. In consequence of the greater breadth of the female pelvis, women are particularly subject to this rupture; insomuch that Mr. Hey never met with any kind of strangulated hernia in females but this.

"The swelling is generally small, and sometimes remarkably so; in the latter case it may easily be mistaken for an inguinal gland slightly enlarged. The circumstances which attended the origin and progress of the tumour, together with its present state and symptoms, generally enable the surgeon to decide upon the nature of the complaint; although the sensible characters of the swelling should be insufficient to lead to this discrimination. The existence of symptoms which usually attend a strangulated hernia, will remove any doubt that the surgeon might entertain on the subject; and if these symptoms do not yield to the usual remedies, will authorize him in operating, although the examination of the tumour should not satisfy his mind that the swelling is a hernia. No great inconvenience can arise from cutting down upon an enlarged gland; while the patient's life would be endangered by putting off the operation in a case of rupture. These considerations would undoubtedly have justified Mr. Else in opening the tumour in the fatal case of crural hernia, which he has recorded in the fourth volume of the Medical Observations and Inquiries; for the want of fecal evacuations clearly pointed out the nature of the affection.

"I have seen a hospital surgeon, a man of considerable practice and eminence in his profession, mistake a femoral hernia for a glandular enlargement, although the attendant symptoms sufficiently indicated the nature of the complaint. So strongly did the tumour in all its sensible characters resemble a swoln gland, that the operation was not performed, although the marks of strangulation were present; and the patient's death afforded an opportunity of ascertaining that the complaint had been caused by a protrusion of the bowel. Similar fatal errors are recorded by Mr. Cooper. The importance of this subject, and the inevitably fatal consequences of a mistake, induce me to repeat, what I have already observed, that the existence of symptoms warrants a surgeon in operating where the symptoms are doubtful. I will venture to add, that, if in compliance with this maxim, the surgeon should, under any unusual concurrence of circumstances, cut down on a merely glandular swelling, he will be acquitted in the opinion of every judicious practitioner; and his conduct will not be attended with any injurious consequence to the patient. If, on the contrary, he persists in preferring the testimony of his touch to the dictates of his reason and judgment, and refuses to operate where the symptoms symptoms demand the use of the knife, he must be considered as responsible for the death of the patient.

"A femoral rupture has often been mistaken for a bubonocele; and the error is not an improbable one, in consequence of the swelling lying, as it frequently does, on Poupart's ligament. The surgeon may consider this mistake as an innocent one, since it does not involve the nature of the complaint, nor the general measures required for its relief. He must change his opinion when he finds that the pressure in the attempts at reduction ought to be exerted in a very different direction; and that the close connexion of various important parts with the crural hernia, would expose him to the risk of some dangerous or even fatal mistake in performing the operation, under such an erroneous idea as to the situation of the rupture. The relation which the neck of the tumour bears to Poupart's ligament, and to the angle of the pubes, will enable the practitioner to distinguish the two cases. If the swelling of a crural hernia be drawn downwards, it will be found that Poupart's ligament can be traced passing over the neck of the sac; while in bubonocele it is found under that part. The angle of the pubes, which is behind and below the neck of the sac in an inguinal hernia, is on the same horizontal level, and rather within it in the crural species."

The anatomical description of the parts follows, which, on account of their more complicated nature, occupies several pages, the author having availed himself of the labours of every writer besides his own very accurate examination. Separate sections follow, on the strangulation, reduction of, and operation for this kind of rupture.

Chapter X. is on umbilical hernia. After describing with much accuracy the sac of this hernia, and adding a few words on the usual contents, the author appropriates distinct sections to the congenital umbilical hernia—the umbilical hernia in young subjects, and the umbilical hernia in adults. The chapter concludes with a few necessary remarks on the operation.

The eleventh and last chapter is on congenital hernia. To the title of this chapter is affixed the following note.

"The term *hernia congenita* was applied to this affection by Haller (*de herniis congenitis*, Gotting. 1749, 4to. *Opuscula patholog.* Lausan. 1755, 8vo.); and the name is sufficiently justifiable, if we consider that the state of parts favouring its occurrence exists at birth, although the rupture itself may not be formed till a subsequent period From this Latin term the English epithet *congenital* has been derived. I cannot understand for what reason Mr. Pott and some others have exchanged this for the appellation *congenial;* which, according to its common use and acceptation, must be perfectly absurd, as applied to this or any other kind of rupture."

After the satisfaction we have received from the perusal of Mr. Lawrence's performance, it would be ungrateful not to tell him all we know on this subject. That Mr. Pott was a scholar, cannot be doubted, but every scholar does not inquire after etymologies, and such seems to have been Mr. Pott's case in this instance. It is well known, that his treatise on that disease was a hasty publication, and having once adopted a word, he might not be willing to relinquish it. But, says our author, "The term is perfectly absurd." It is so; and we do not recollect any *original* writer but Mr. Pott who has used it. However, he does not appear to be the father of so absurd a brat: For though we suspect that the first edition of Mr. Pott's *account of a particular kind of rupture*, is of an earlier date than is here assigned to it, yet it was posterior to a translation of Haller's Opuscula Pathologica, which was published by Wilson and Denham, in the year 1756. The translation is anonymous, and by the above expression, and many other marks, appears not to have been the performance of a medical writer.

The chapter on congenital hernia is like the rest of the work, well written, though it appears short. The author has, indeed, referred to a great number of authorities, and it must be admitted that the dissecting room is the properest place to demonstrate the journey of the testicles *.

We here take our leave of this truly useful performance, which we conceive it must be unnecessary to recommend, as it will doubtless form a part of every medical library. An interesting case of artificial anus, is contained in the appendix, told with much more minuteness than we have ever seen before.

* *Testium iter*, is an expression of Haller's.

